# Association of triglyceride glucose body mass index with osteoporosis risks in a large Chinese adult cohort

**DOI:** 10.3389/fendo.2025.1680775

**Published:** 2025-09-23

**Authors:** Hongshun Liu, Hongyu Tan, Yao Pan

**Affiliations:** ^1^ Department of Orthopedics, Zhengzhou Orthopedic Hospital, Henan, China; ^2^ Tieling Health Vocational College, Liaoning, China; ^3^ Department of Orthopedics, The Sixth People’s Hospital Affiliated to Shanghai Jiao Tong University School of Medicine, Shanghai, China

**Keywords:** TyG-BMI(triglyceride glucose-body mass index), osteoporosis, cohort study, electronic health record - (EHR), Chinese population

## Abstract

**Background:**

Insulin resistance (IR) is implicated in bone metabolism dysregulation, but the role of Triglyceride Glucose Body Mass Index (TyG-BMI), a surrogate marker integrating lipid-glucose metabolism and adiposity, in osteoporosis risk remains underexplored.

**Methods:**

We analyzed 23,930 participants categorized into TyG-BMI quartiles (Q1–Q4). Baseline characteristics, biochemical profiles, and medication use were compared. Osteoporosis incidence was tracked over a 4-year median follow-up. Cox models estimated hazard ratios (HRs) for osteoporosis across quartiles of TyG-BMI, adjusted for confounders. Stratified analyses were performed to explore the effect modification by age, sex, renal function, smoking, and medication use.

**Results:**

Osteoporosis incidence (1,134 cases) rose sharply from Q1 (71 cases) to Q4 (855 cases).Participants in Q4 (highest TyG-BMI) were younger (mean age 56.2 *vs*. Q1: 63.0 years) with higher BMI (29.7 *vs*. 20.8 kg/m²), fasting glucose (9.15 *vs*. 7.02 mmol/L), triglycerides (2.95 *vs*. 1.06 mmol/L). Osteoporosis incidence rose sharply from Q1 (71 cases) to Q4 (855 cases). In fully-adjusted models, Q4 had a 3.67-fold higher osteoporosis risk *vs*. Q1 (95% CI: 2.80–4.80; P < 0.001). Stratified analyses revealed stronger associations in participants aged <65 years (HR: 14.6; 95% CI: 10.4–20.6), males (HR: 12.4; 95% CI: 8.79–17.6), smokers (HR: 15.2; 95% CI: 6.77–34.0), and those with preserved renal function (HR: 12.2; 95% CI: 8.99–16.7).

**Conclusion:**

Elevated TyG-BMI independently predicts incident osteoporosis, with the highest risk in younger males, smokers, and individuals with preserved renal function. TyG-BMI may serve as a practical tool for early osteoporosis risk stratification.

## Introduction

Osteoporosis, a systemic skeletal disorder characterized by compromised bone strength and increased fracture risk, poses a growing global health burden, with over 200 million affected individuals and an estimated economic cost exceeding $100 billion annually (1, 2). While traditional risk factors such as advanced age, female sex, and postmenopausal status are well-established (3, 4), emerging evidence implicates metabolic dysregulation, particularly insulin resistance (IR) and chronic inflammation, as critical drivers of bone loss (5–8). IR disrupts bone homeostasis through multiple pathways including impaired osteoblast differentiation via suppressed insulin-like growth factor 1 (IGF-1) signaling, enhanced osteoclast activity through upregulated receptor activator of nuclear factor kappa-B ligand (RANKL) and altered adipokine secretion from visceral fat (9, 10).

The Triglyceride-Glucose Index (TyG), calculated from fasting triglycerides and glucose, is a validated surrogate marker of IR (11). Its integration with body mass index (BMI), yielding the TyG-BMI, creates a composite metric that captures both metabolic disorders (including hypertriglyceridemia and hyperglycemia) and obesity (BMI), offering superior predictive value for cardiometabolic diseases compared to TyG or BMI alone (12, 13). Recent studies link TyG-BMI to incident diabetes, cardiovascular events, and chronic kidney disease (14–16). Furthermore, a limited number of recent studies have directly investigated TyG-BMI in relation to bone mineral density (BMD) and osteoporosis, but findings appear context-dependent.

While obesity traditionally confers mechanical loading benefits to bone, visceral adiposity in metabolic obesity promotes inflammation and marrow adipogenesis, directly inhibiting osteogenesis (17). Given that osteoporosis in the context of metabolic dysfunction is likely driven by the dual insult of impaired glucose-lipid metabolism and adverse adipokine profiles from visceral fat, the TyG-BMI, which synergistically captures lipid-glucose dysregulation and adiposity, may serve as a more holistic predictor of osteoporosis risk than its individual components, particularly in populations with underlying metabolic dysfunction. This study aimed to evaluate the relationship between TyG-BMI and incident osteoporosis in a large real-world Chinese cohort and to identify high-risk subgroups through stratified analyses.

## Methods

### Study design and participants

**Figure 1:**
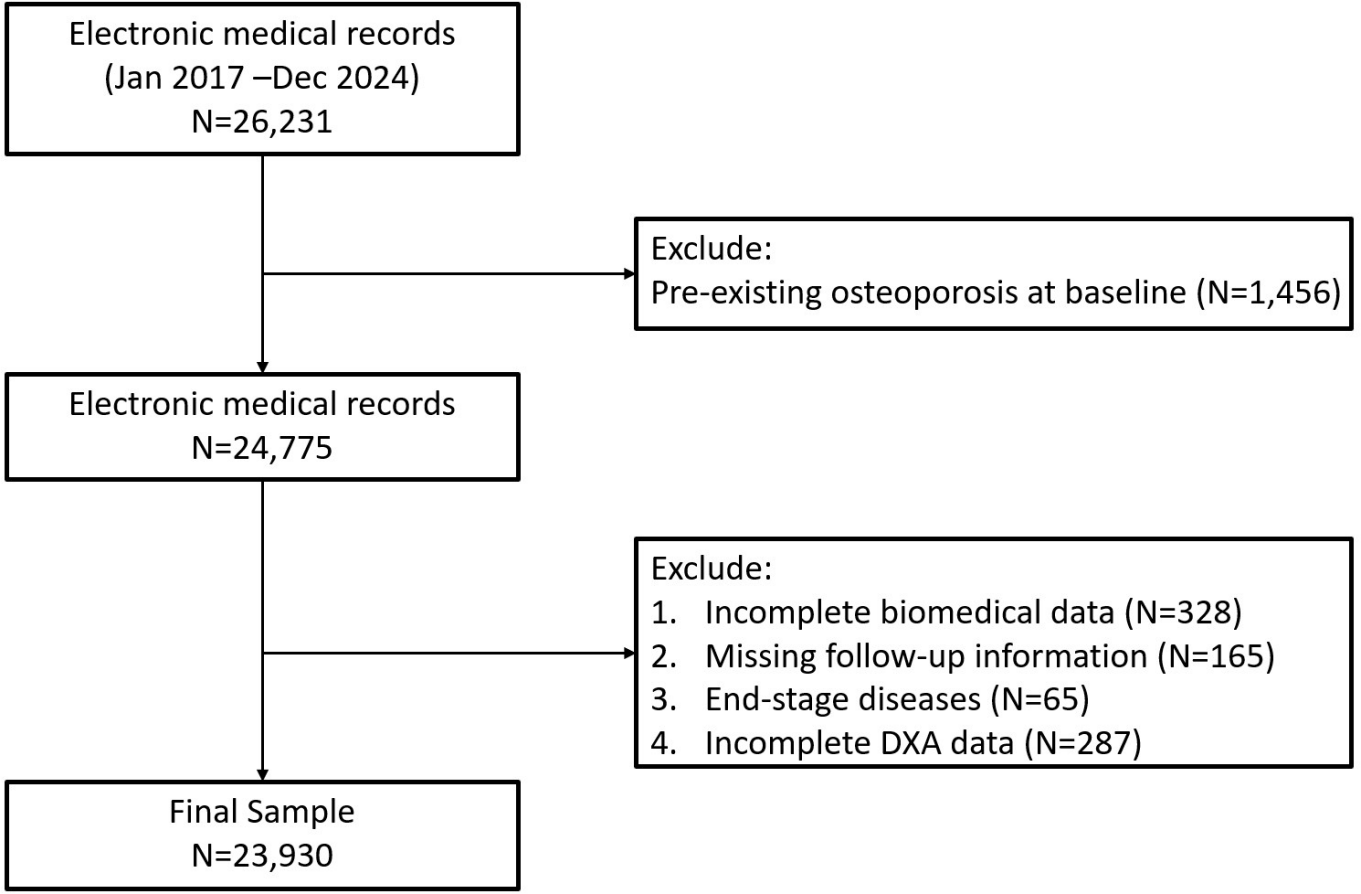
Flow chart of study population.

This study was a real-world, retrospective cohort analysis conducted using electronic health records (EHR) from the Department of Orthopedics, Shanghai Sixth People’s Hospital Affiliated to Shanghai Jiao Tong University School of Medicine. Participants included were those who had outpatient or inpatient visits between January 2017 and December 2024. The study population initially comprised 23,930 adults aged ≥18 years with complete clinical records necessary for calculating the triglyceride-glucose body mass index (TyG-BMI). Individuals with pre-existing osteoporosis at baseline, incomplete biochemical data, missing follow-up information including DXA or end-stage diseases were excluded from the study (**Figure 1**, **Supplementary Table 1**). The final cohort was categorized into quartiles (Q1-Q4) based on baseline TyG-BMI values. This study was approved by the Institution Review Board (IRB) of Shanghai Sixth People’s Hospital Affiliated to Shanghai Jiao Tong University School of Medicine (IRB number 2024-KY-163 (K)). Informed consents were waived since we used deidentified data.

### Data collection

Baseline demographic data, clinical parameters, biochemical profiles, medication history, and lifestyle factors including smoking status were collected from the hospital’s EHR system. BMI was calculated as weight in kilograms divided by the square of height in meters (kg/m²). The TyG-BMI was computed as follows: TyG-BMI = ln(fasting triglycerides [mg/dL] × fasting plasma glucose [mg/dL]/2) × BMI.

Biochemical parameters assessed included fasting plasma glucose, triglycerides, glycated hemoglobin (HbA1c), lipid profiles (HDL-cholesterol, LDL-cholesterol, total cholesterol), and renal function evaluated by estimated glomerular filtration rate (eGFR). Medication data encompassed usage of antidiabetic drugs (metformin, SGLT2 inhibitors, GLP-1 receptor agonists), lipid-lowering medications, and antihypertensive agents.

### Outcome measures

The primary outcome was incident osteoporosis, identified using dual-energy X-ray absorptiometry (DXA) scans according to the World Health Organization (WHO) criteria (T-score ≤ -2.5 at lumbar spine, femoral neck, or total hip), which further confirmed by the physicians by International Classification of Diseases, 10th Revision (ICD-10) codes M81. Both criteria were required: a DXA T−score ≤ −2.5 at the lumbar spine, femoral neck, or total hip and a physician-confirmed ICD−10 M81 entry in the EHR within a prespecified window. Follow-up continued until the occurrence of osteoporosis, loss to follow-up, death, or end of the study period (December 2022), whichever occurred first. The median follow-up duration was approximately 4 years.

### Statistical analysis

Baseline characteristics were compared across TyG-BMI quartiles using ANOVA or Kruskal-Wallis tests for continuous variables and chi-square tests for categorical variables. The incidence rates of osteoporosis were calculated per quartile. Cox proportional hazards regression models were employed to estimate HRs and 95% confidence intervals (CIs) for osteoporosis risk across TyG-BMI quartiles, adjusting for age, sex, smoking status, smoking status, eGFR category, HDL−C, LDL−C, SBP, HbA1c, antihypertensive use, lipid−lowering therapy, and glucose−lowering therapy. We also performed variance inflation factors to ensure no collinearity among covariates. To complement the Cox models, we have added the Kaplan–Meier curves by TyG−BMI quartile with log−rank tests. The lowest quartile (Q1) served as the reference group. Subgroup analyses were conducted stratified by age (<65 *vs*. ≥65 years), sex, renal function (eGFR categories), smoking status, and medication use to explore potential effect modifications. Interaction terms were tested to determine statistically significant differences between subgroups. For Cox model assumptions and diagnostics, we assessed the proportional hazards (PH) assumption using (i) global and covariate−specific scaled Schoenfeld residual tests, and (ii) visual inspection of log(−log) survival curves across TyG−BMI quartiles. When a covariate suggested PH violation (p<0.05 or systematic divergence on plots), we implemented (a) time−by−covariate interaction terms to allow time−varying effects, or (b) stratified Cox models (strata variables not estimated but allowed to have separate baselines), as appropriate. We evaluated influential observations using dfbeta and deviance residuals, and we report models with and without influential points as a robustness check. Although quartiles of TyG−BMI were the primary exposure for interpretability, we also examined TyG−BMI as a continuous variable using restricted cubic splines (3–4 knots) to assess potential nonlinearity; spline plots and Wald tests are provided in the Supplementary. To guard against mild model misspecification, we provide robust (sandwich) standard errors. For missing data, we characterized missingness patterns for covariates and laboratory values at baseline. The primary analysis used multiple imputation by chained equations (MICE) for covariates with missingness <25%, generating m=20 imputed datasets that included all adjustment variables, the event indicator, and the log of follow−up time. Continuous variables were imputed via predictive mean matching (k=5), binary variables via logistic regression, and ordinal variables via proportional odds models; imputations respected clinically plausible bounds. We combined estimates across imputations using Rubin’s rules. Statistical significance was set at a two-tailed p-value <0.05. All statistical analyses were performed using R statistical software (version 4.2.0, R Foundation for Statistical Computing, Vienna, Austria).

## Results

### Participant selection and baseline characteristics

A total of 23930 participants were categorized into quartiles based on TyG-BMI levels (**Table 1**). As TyG-BMI increased from Q1 to Q4, several notable trends in baseline characteristics emerged. Participants in the highest quartile (Q4) were younger (mean age: 56.2 years) compared to those in Q1 (63.0 years), with a corresponding increase in mean BMI from 20.8 kg/m² in Q1 to 29.7 kg/m² in Q4 (both P<0.01). The proportion of male participants was relatively balanced across quartiles, ranging from 55.8% in Q1 to 60.1% in Q3 (P>0.05). Blood pressure remained relatively stable across quartiles, with mean systolic pressure approximately 129 mmHg and diastolic pressure slightly increasing from 72.3 mmHg in Q1 to 73.6 mmHg in Q4. Fasting plasma glucose levels rose progressively with TyG-BMI, from 7.02 mmol/L in Q1 to 9.15 mmol/L in Q4. Renal function, as measured by eGFR, was slightly higher in Q4 (91.6 mL/min/1.73m²) compared to Q1 (87.5 mL/min/1.73m²). Lipid profiles showed a worsening pattern with increasing TyG-BMI: triglyceride levels increased from 1.06 mmol/L in Q1 to 2.95 mmol/L in Q4, while HDL-cholesterol decreased from 1.21 mmol/L to 0.98 mmol/L. LDL-cholesterol and total cholesterol levels also rose across quartiles.

**Table 1:** Baseline characteristics.

Variables	TyG-BMI			
	Q1 ≤198	Q2 >198 to≤222	Q3 >222to≤251	Q4 >251
Age(years)	63.0 (14.4)	63.1 (12.0)	61.3 (12.1)	56.2 (14.4)
BMI (kg/m^2^)	20.8 (2.03)	23.6 (1.46)	25.6 (1.67)	29.7 (4.25)
Male (n, %)	3338(55.8%)	3571(59.7%)	3596(60.1%)	3464(57.9%)
Systolic blood pressure (mmHg)	129 (18.7)	129 (18.6)	129 (19.3)	129 (18.8)
Diastolic blood pressure (mmHg)	72.3 (12.5)	72.9 (13.2)	73.3 (13.4)	73.6 (13.6)
Fasting plasma glucose (mmol/L)*	7.02 (2.73)	7.70 (2.86)	8.27 (3.06)	9.15 (3.39)
HbA1c (%)	5.69 (1.28)	5.69 (1.30)	5.67 (1.25)	5.69 (1.29)
eGFR	87.5 (30.2)	86.4 (28.5)	88.3 (27.5)	91.6 (30.6)
LDL-cholesterol (mmol/L)	2.56 (0.94)	2.75 (0.95)	2.86 (1.01)	2.86 (0.99)
Triglycerides (mmol/L)*	1.06 (0.58)	1.45 (0.86)	1.88 (1.22)	2.95 (3.16)
HDL-cholesterol (mmol/L)	1.21 (0.39)	1.08 (0.31)	1.02 (0.28)	0.98 (0.27)
Total cholesterol (mmol/L)	4.35 (1.15)	4.52 (1.17)	4.71 (1.23)	5.03 (1.45)
Anti-diabetes medications (n, %)	2537 (42.4%)	2543 (42.5%)	2530 (42.3%)	2614 (43.7%)
Metformin (n, %)	2321 (38.8%)	2280 (38.1%)	2232 (37.3%)	2310 (38.6%)
DPP4i (n, %)	179 (3.0%)	203 (3.4%)	203 (3.4%)	221 (3.7%)
SGLT2i (n, %)	18 (0.3%)	30 (0.5%)	36 (0.6%)	54 (0.9%)
TZD (n, %)	36 (0.6%)	54 (0.9%)	54 (0.9%)	96 (1.6%)
GLP-1RA (n, %)	251 (4.2%)	377 (6.3%)	443 (7.4%)	508 (8.5%)
Insulin (n, %)	233 (3.9%)	317 (5.3%)	287 (4.8%)	299 (5.0%)
Lipid-lowering medications (n, %)	497 (8.3%)	580 (9.7%)	574 (9.6%)	586 (9.8%)
Anti-hypertension medications (n, %)	556 (9.3%)	580 (9.7%)	598 (10.0%)	604 (10.1%)
Smoking (n, %)	102 (1.7%)	54 (0.9%)	544 (9.1%)	2470 (41.3%)

*All mmol/L values were converted to mg/dL prior to calculating TyG−BMI (FPG: mmol/L × 18; TG: mmol/L × 88.57).

The proportion of participants using anti-diabetic medications was similar across groups (~42%), and the use of metformin ranked the first and was balanced across quartiles. The use of lipid-lowering and antihypertensive medications remained relatively constant across quartiles. Notably, smoking prevalence increased markedly from 1.7% in Q1 to 41.3% in Q4.

### Primary association analysis

During a median follow-up of approximately 4 years, a total of 1,134 incident osteoporosis cases were identified among 23,930 participants. The incidence of osteoporosis increased markedly across TyG-BMI quartiles, from 71 cases in Q1 to 855 in Q4. In the unadjusted model, participants in the highest TyG-BMI quartile (Q4) had a substantially elevated risk of developing osteoporosis compared to those in the lowest quartile (Q1), with a hazard ratio (HR) of 11.0 (95% CI: 8.62–14.0, P < 0.001). After adjusting for potential confounders, the association remained significant though attenuated, with an adjusted HR of 3.67 (95% CI: 2.80–4.80, P < 0.001).

**Figure 2:**
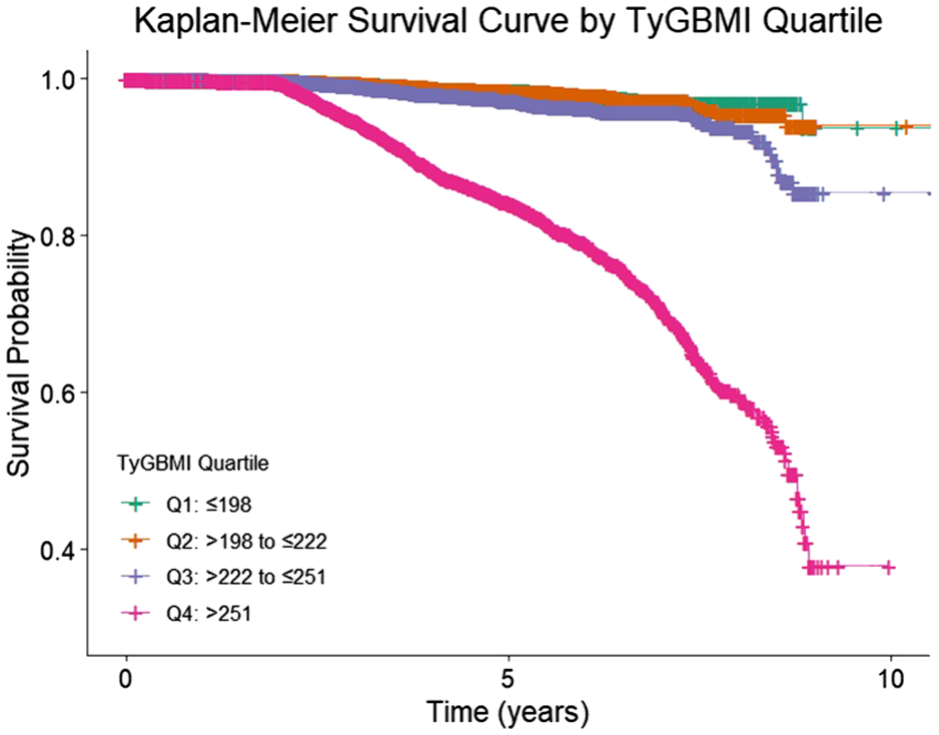
KM survival curve of TyG-BMI (quartile).

In contrast, the middle quartiles showed weaker and non-significant associations after adjustment. Specifically, compared with Q1, the adjusted HR was 0.81 (95% CI: 0.58–1.12) for Q2 and 1.13 (95% CI: 0.84–1.51) for Q3. These findings suggest a strong, graded relationship between higher TyG-BMI and increased risk of osteoporosis, primarily driven by the highest quartile (**Table 2**, **Figure 2**). We performed sensitivity analysis to exclude participants who had been diagnosed as osteoporosis at the first six months of follow-up. We also examined TyG−BMI as a continuous variable using restricted cubic splines (4 knots) to assess potential nonlinearity. (**Supplementary Table 2**, **Supplementary Figure 1**).

**Table 2:** Hazard ratios of TyG-BMI with incident osteoporosis risks.

	No. of participants	No. of cases	Mean follow-up years	Crude model	Multiple variable adjusted model*
TyG-BMI					
Q1	5983	71	3.79 (1.99)	1.00	1.00
Q2	5983	75	3.91 (1.93)	1.02 (0.73, 1.41)	0.81 (0.58, 1.12)
Q3	5982	133	4 (1.91)	1.75 (1.31, 2.33)	1.13 (0.84, 1.51)
Q4	5982	855	4.1 (1.83)	11.0 (8.62, 14.0)	3.67 (2.80, 4.80)

*The model was adjusted for age, sex, smoking status, smoking status, eGFR category, HDL−C, LDL−C, SBP, HbA1c, antihypertensive use, lipid−lowering therapy, and glucose−lowering therapy.

### Subgroup and interaction analyses

Stratified analyses revealed that the positive association between higher TyG-BMI and incident osteoporosis remained robust across most subgroups, though the magnitude of association varied (**Table 3**). A statistically significant interaction by age was observed (P for interaction = 0.048), with the association being stronger among participants aged <65 years (adjusted HR for Q4 *vs*. Q1: 14.6; 95% CI: 10.4–20.6; P < 0.001) compared to those aged ≥65 years (HR: 6.30; 95% CI: 4.41–9.02; P < 0.001).

**Table 3:** Subgroup analysis.

	TyG-BMI				As a continuous variable	P for interaction
	Q1	Q2	Q3	Q4		
Age, years old						0.048
<65	1	0.92 (0.57, 1.49)	2.12 (1.43, 3.16)	14.6 (10.4, 20.6)	1.01 (1.01, 1.02)	
≥65	1	1.13 (0.72, 1.75)	1.41 (0.92, 2.17)	6.30 (4.41, 9.02)	1.01 (1.00, 1.02)	
Sex						<0.001
Male	1	0.99 (0.63, 1.57)	1.66 (1.10, 2.51)	12.4 (8.79, 17.6)	1.02 (1.02, 1.02)	
Female	1	1.07 (0.67, 1.69)	1.89 (1.27, 2.84)	9.55 (6.80, 13.4)	1.01 (1.01, 1.01)	
Estimated GFR, mL/min/1.73 m^2^						<0.001
≥90	1	1.04 (0.68, 1.58)	1.79 (1.23, 2.59)	12.2 (8.99, 16.7)	1.01 (1.01, 1.02)	
60-89	1	1.01 (0.51, 2.04)	1.75 (0.94, 3.24)	9.52 (5.59, 16.2)	1.01 (1.01, 1.01)	
<60	1	0.89 (0.42, 1.90)	1.66 (0.84, 3.27)	8.33 (4.70, 14.7)	1.01 (1.01, 1.01)	
Smoking status						0.001
Current and past smoking	–	–	1	15.2 (6.77, 34.0)	1.01 (1.01, 1.02)	
Never smoking	1	1.00 (0.73, 1.39)	1.82 (1.36, 2.43)	10.1 (7.83, 12.9)	1.01 (1.01, 1.01)	
Lipid-lowering medications						<0.001
No use	1	1.00 (0.71, 1.40)	1.69 (1.25, 2.29)	10.7 (8.34, 13.8)	1.01 (1.01, 1.01)	
Use	1	1.17 (0.41, 3.37)	2.24 (0.88, 5.73)	12.9 (5.66, 29.5)	1.02 (1.01, 1.02)	
Antihypertensive medications						<0.001
No use	1	1.06 (0.75, 1.49)	1.78 (1.31, 2.42)	11.2 (8.64, 14.5)	1.01 (1.01, 1.01)	
Use	1	0.75 (0.28, 2.02)	1.48 (0.65, 3.39)	9.23 (4.66, 18.3)	1.02 (1.01, 1.02)	
Glucose-lowering medications		0.86 (0.54, 1.36)	1.47 (0.98, 2.20)	9.05 (6.51, 12.6)	1.02 (1.02, 1.02)	<0.001
Metformin	1	0.88 (0.55, 1.42)	1.35 (0.88, 2.06)	8.68 (6.16, 12.2)	1.02 (1.02, 1.02)	<0.001
DPP4i	–	1	3.29 (0.66, 16.3)	12.8 (3.05, 53.8)	1.01 (1.00, 1.01)	0.695
SGLT2i	–	–	1	11.9 (1.58, 89.4)	1.02 (1.00, 1.02)	0.9903
TZD	1	1.43 (0.13, 15.8)	1.26 (0.11, 13.9)	5.67 (0.74, 43.3)	1.02 (1.01, 1.03)	0.0385
GLP-1RA	1	1.60 (0.31, 8.24)	3.90 (0.89, 17.1)	18.8 (4.62, 76.4)	1.01 (1.01, 1.02)	0.1673
Insulin	1	0.28 (0.05, 1.45)	1.14 (0.36, 3.59)	5.66 (2.23, 14.5)	1.01 (1.00, 1.01)	0.786

Sex-stratified analysis showed that the association was significant in both males and females but was more pronounced in males (Q4 *vs*. Q1 HR: 12.4; 95% CI: 8.79–17.6) than in females (HR: 9.55; 95% CI: 6.80–13.4), with a significant interaction (P < 0.001). A consistent trend was also observed across different levels of renal function, with a stronger association among participants with eGFR ≥90 mL/min/1.73 m² (HR: 12.2; 95% CI: 8.99–16.7), though interaction by eGFR was also significant (P < 0.001).

Smoking status modified the association (P for interaction = 0.001). Among never smokers, the adjusted HR for Q4 *vs*. Q1 was 10.1 (95% CI: 7.83–12.9), while among current and former smokers, the HR was even higher at 15.2 (95% CI: 6.77–34.0). Similarly, the effect of TyG-BMI on osteoporosis risk was stronger among individuals not taking lipid-lowering or antihypertensive medications, with significant interactions observed for both (both P < 0.001).

Subgroup analyses by use of glucose-lowering agents indicated significant interactions for several medication classes. The association remained evident among users of metformin (HR for Q4 *vs*. Q1: 8.68; 95% CI: 6.16–12.2; P for interaction < 0.001), and among GLP-1 RA users, where the HR was markedly elevated (18.8; 95% CI: 4.62–76.4), although this did not reach statistical significance (P = 0.1673). Notably, the association was also strong in users of DPP-4 inhibitors, SGLT2 inhibitors, and TZDs, but wide confidence intervals suggest limited precision due to small sample sizes.

## Discussion

This large cohort study established TyG-BMI as a novel, independent predictor of incident osteoporosis, with a striking 3.67-fold elevated risk in the highest versus lowest quartile after adjustment. Our findings extended prior work on IR and bone health by demonstrating that a composite metric integrating lipid-glucose dysregulation and adiposity outperforms individual components in osteoporosis prediction, particularly among younger males, smokers, and those with preserved renal function, suggests that TyG-BMI, a simple and cost-effective composite index, could be integrated into routine health assessments as a practical screening tool.

Insulin resistance, characterized by diminished responsiveness of peripheral tissues to insulin, is a hallmark of metabolic syndrome and an emerging risk factor for bone BMD loss (18). Paradoxically, insulin exerts anabolic effects on bone by promoting osteoblast proliferation and survival, thereby increasing bone mass. Given that both insulin resistance and compensatory hyperinsulinemia are central to type 2 diabetes pathogenesis, elevated bone mass might be anticipated in diabetic patients (19). Consequently, IR likely exerts dual opposing effects on skeletal integrity including impaired insulin signaling in osteoblasts, suppresses their activity, reducing bone formation (20) and Concurrent hyperinsulinemia may partially counteract this through direct trophic actions.

The pathogenesis of osteoporosis involves multifactorial mechanisms, with key contributors including advanced age, obesity, hyperinsulinemia, hyperglycemia, metabolic syndrome, alterations in gastrointestinal hormones, and renal dysfunction (21–23). Osteoporotic fractures, the most severe complication, predominantly affect vertebral bodies, hips, distal radii, and proximal humeri (24). In advanced disease, vertebral compression fractures precipitate height loss and kyphotic deformity, while the compromised bone quality exacerbates mechanical instability. This not only complicates fracture healing but also adversely affects surgical outcomes in spinal interventions (25).

Our findings align with prior studies investigating the relationship between insulin resistance surrogates and osteoporosis. In a cohort of type 2 diabetes mellitus (T2DM) patients, Zhan et al. reported that the TyG index (without BMI integration) independently predicted osteoporosis with an AUC of 0.857 when combined with age and sex (26). Similarly, Chen et al. demonstrated that TyG-BMI was a robust risk factor for osteoporosis in middle-aged and elderly T2DM patients, exhibiting a threshold effect at TyG-BMI >191.52 (27). Consistent with these studies, we observed a strong, graded association between elevated TyG-BMI and incident osteoporosis, with a 3.67-fold increased risk in the highest versus lowest quartile. Notably, our threshold effect analysis revealed a sharp risk escalation in Q4, suggesting a critical TyG-BMI level beyond which bone loss accelerates.

However, contrasting results exist in non-diabetic populations. Tian et al. found that higher TyG-BMI was positively correlated with BMD and associated with lower osteoporosis risk among US adults without diabetes (28). This divergence may reflect the ‘obesity paradox’ in bone health: whereas moderate adiposity may confer mechanical benefits to bone in metabolically healthy individuals, the confluence of hyperglycemia, dyslipidemia, and insulin resistance in prediabetic/diabetic states promotes inflammation, oxidative stress, and AGE accumulation, ultimately compromising bone quality (29). Our cohort, characterized by elevated fasting glucose (7.02–9.15 mmol/L across quartiles), thereby accentuating the detrimental metabolic effects of high TyG-BMI.

Beyond confirming TyG-BMI as a predictor, our study identified high-risk subgroups. The association was markedly stronger in males (HR 12.4 *vs*. 9.55 in females), current/former smokers (HR 15.2), and those with preserved renal function (eGFR ≥90 mL/min/1.73m²; HR 12.2). This aligns with Chen et al.’s gender-stratified thresholds (2) but extends to modifiable risk factors like smoking, which may synergize with insulin resistance via oxidative pathways. These insights enable precision screening., targeting young male smokers with TyG-BMI > [Q4 cutoff] for DXA scans.

This real−world, single−health−system EHR cohort is subject to several limitations that warrant consideration. First, misclassification of outcomes and exposures is possible because osteoporosis was ascertained from DXA T−scores with corroborating ICD−10 M81 codes and TyG−BMI was derived from routine fasting laboratories; to balance this risk we performed sensitivity analyses restricting the endpoint to DXA−confirmed events only, requiring baseline and follow−up DXA in a restricted cohort to reduce surveillance bias, and using the earliest fasting values on the index date to minimize exposure misclassification, with results remaining directionally consistent. Second, residual confounding may persist despite multivariable adjustment, including unmeasured factors such as diet, calcium/vitamin D supplementation, physical activity, socioeconomic determinants of health, and confounding by indication related to medication use; for balance, we added models that further adjusted for HDL−C and eGFR categories, excluded events within 6 months of index (to mitigate reverse causation), and repeated the analysis as a complete−case set alongside multiple imputation by chained equations. Third, informative censoring and loss to follow−up could bias estimates; we therefore summarized attrition and applied inverse probability of censoring weights, which yielded similar inferences. Finally, generalizability may be limited because testing practices, DXA utilization, and case−mix can differ across health systems; replication in external datasets and prospective settings will be important to confirm portability of the findings. Collectively, these diagnostics and sensitivity analyses support the robustness of the observed associations while acknowledging the inherent constraints of EHR−based research.

## Conclusion

TyG-BMI is a robust, accessible predictor of osteoporosis, particularly in young males, smokers, and those with preserved renal function. Its integration into clinical practice could enhance early risk identification, enabling targeted interventions in high-risk populations. Future studies should validate TyG-BMI thresholds for osteoporosis screening in diverse populations and explore targeted interventions for insulin-resistant individuals.

## Data Availability

The data used in this study are not publicly available. However, they may be provided upon reasonable request to the corresponding author. Requests to access these datasets should be directed to Yao Pan: py6thnagaqueen@163.com.

## References

[1] Cosman F Lewiecki EM Eastell R Ebeling PR Jan De Beur S Langdahl B. Goal-directed osteoporosis treatment: ASBMR/BHOF task force position statement 2024. J Bone Miner Res. (2024) 39:1393–405. doi: 10.1093/jbmr/zjae119, PMID: 39073912 PMC11425703

[2] GBD 2019 Fracture Collaborators. Global, regional, and national burden of bone fractures in 204 countries and territories, 1990-2019: a systematic analysis from the Global Burden of Disease Study 2019. Lancet Healthy Longev. (2021) 2:e580–92. doi: 10.1016/S2666-7568(21)00172-0, PMID: 34723233 PMC8547262

[3] Händel MN Cardoso I von Bülow C Rohde JF Ussing A Nielsen SM. Fracture risk reduction and safety by osteoporosis treatment compared with placebo or active comparator in postmenopausal women: systematic review, network meta-analysis, and meta-regression analysis of randomised clinical trials. BMJ. (2023) 381:e068033. doi: 10.1136/bmj-2021-068033, PMID: 37130601 PMC10152340

[4] Ayers C Kansagara D Lazur B Fu R Kwon A Harrod C. Effectiveness and safety of treatments to prevent fractures in people with low bone mass or primary osteoporosis: A living systematic review and network meta-analysis for the american college of physicians. Ann Intern Med. (2023) 176:182–95. doi: 10.7326/M22-0684, PMID: 36592455

[5] Fu YH Liu WJ Lee CL Wang JS. Associations of insulin resistance and insulin secretion with bone mineral density and osteoporosis in a general population. Front Endocrinol (Lausanne). (2022), 13: 971960. doi: 10.3389/fendo.2022.971960, PMID: 36204101 PMC9530363

[6] Armutcu F McCloskey E. Insulin resistance, bone health, and fracture risk. Osteoporos Int. (2024) 35:1909–17. doi: 10.1007/s00198-024-07227-w, PMID: 39264439

[7] Kanno Y. The roles of fibrinolytic factors in bone destruction caused by inflammation. Cells. (2024) 13:516. doi: 10.3390/cells13060516, PMID: 38534360 PMC10968824

[8] Turner JD Naylor AJ Buckley C Filer A Tak PP. Fibroblasts and osteoblasts in inflammation and bone damage. Adv Exp Med Biol. (2018) 1060:37–54. doi: 10.1007/978-3-319-78127-3_3, PMID: 30155621

[9] Contreras-Bolívar V García-Fontana B García-Fontana C Muñoz-Torres M. Mechanisms involved in the relationship between vitamin D and insulin resistance: impact on clinical practice. Nutrients. (2021) 13:3491. doi: 10.3390/nu13103491, PMID: 34684492 PMC8539968

[10] Shirinezhad A Azarboo A Ghaseminejad-Raeini A Kanaani Nejad F Zareshahi N Amiri SM. A systematic review of the association between insulin resistance surrogate indices and bone mineral density. Front Endocrinol (Lausanne). (2024) 15:1499479. doi: 10.3389/fendo.2024.1499479, PMID: 39744182 PMC11688183

[11] Dang K Wang X Hu J Zhang Y Cheng L Qi X. The association between triglyceride-glucose index and its combination with obesity indicators and cardiovascular disease: NHANES 2003-2018. Cardiovasc Diabetol. (2024) 23:8. doi: 10.1186/s12933-023-02115-9, PMID: 38184598 PMC10771672

[12] Zhou Z Liu Q Zheng M Zuo Z Zhang G Shi R. Comparative study on the predictive value of TG/HDL-C, TyG and TyG-BMI indices for 5-year mortality in critically ill patients with chronic heart failure: a retrospective study. Cardiovasc Diabetol. (2024) 23:213. doi: 10.1186/s12933-024-02308-w, PMID: 38902757 PMC11191322

[13] Li W Shen C Kong W Zhou X Fan H Zhang Y. Association between the triglyceride glucose-body mass index and future cardiovascular disease risk in a population with Cardiovascular-Kidney-Metabolic syndrome stage 0-3: a nationwide prospective cohort study. Cardiovasc Diabetol. (2024) 23:292. doi: 10.1186/s12933-024-02352-6, PMID: 39113004 PMC11308445

[14] Shao Y Hu H Li Q Cao C Liu D Han Y. Link between triglyceride-glucose-body mass index and future stroke risk in middle-aged and elderly chinese: a nationwide prospective cohort study. Cardiovasc Diabetol. (2024) 23:81. doi: 10.1186/s12933-024-02165-7, PMID: 38402161 PMC10893757

[15] Huang D Ma R Zhong X Jiang Y Lu J Li Y. Positive association between different triglyceride glucose index-related indicators and psoriasis: evidence from NHANES. Front Immunol. (2023) 14:1325557. doi: 10.3389/fimmu.2023.1325557, PMID: 38179048 PMC10765499

[16] Dou J Guo C Wang Y Peng Z Wu R Li Q. Association between triglyceride glucose-body mass and one-year all-cause mortality of patients with heart failure: a retrospective study utilizing the MIMIC-IV database. Cardiovasc Diabetol. (2023) 22:309. doi: 10.1186/s12933-023-02047-4, PMID: 37940979 PMC10634170

[17] Gkastaris K Goulis DG Potoupnis M Anastasilakis AD Kapetanos G. Obesity, osteoporosis and bone metabolism. J Musculoskelet Neuronal Interact. (2020) 20:372–81., PMID: 32877973 PMC7493444

[18] Li J Ma C Wang X Li J Liu P Zhu M. Development and validation of a novel glucolipid metabolism-related nomogram to enhance the predictive performance for osteoporosis complications in prediabetic and diabetic patients. Lipids Health Dis. (2025) 24:183. doi: 10.1186/s12944-025-02602-w, PMID: 40399876 PMC12093595

[19] Jia F Lu Y Wen H Tu J Ning X Wang J. Correlations between tyG-related indices and bone health: A cross-sectional study of osteoporosis in a rural chinese population. Diabetes Metab Syndr Obes. (2025) 18:1445–58. doi: 10.2147/DMSO.S505024, PMID: 40356712 PMC12067712

[20] Wang LT Chen LR Chen KH. Hormone-related and drug-induced osteoporosis: A cellular and molecular overview. Int J Mol Sci. (2023) 24:5814. doi: 10.3390/ijms24065814, PMID: 36982891 PMC10054048

[21] Lane NE. Epidemiology, etiology, and diagnosis of osteoporosis. Am J Obstet Gynecol. (2006) 194:S3–11. doi: 10.1016/j.ajog.2005.08.047, PMID: 16448873

[22] Subarajan P Arceo-Mendoza RM Camacho PM. Postmenopausal osteoporosis: A review of latest guidelines. Endocrinol Metab Clin North Am. (2024) 53:497–512. doi: 10.1016/j.ecl.2024.08.008, PMID: 39448132

[23] Muñoz M Robinson K Shibli-Rahhal A. Bone health and osteoporosis prevention and treatment. Clin Obstet Gynecol. (2020) 63:770–87. doi: 10.1097/GRF.0000000000000572, PMID: 33017332

[24] Kendler DL Marin F Zerbini CAF Russo LA Greenspan SL Zikan V. Effects of teriparatide and risedronate on new fractures in post-menopausal women with severe osteoporosis (VERO): a multicentre, double-blind, double-dummy, randomised controlled trial. Lancet. (2018) 391:230–40. doi: 10.1016/S0140-6736(17)32137-2, PMID: 29129436

[25] Li C Lai XM Liu N Lin Y Hu W. Correlation analysis of the vertebral compression degree and CT HU value in elderly patients with osteoporotic thoracolumbar fractures. J Orthop Surg Res. (2023) 18:457. doi: 10.1186/s13018-023-03941-z, PMID: 37365576 PMC10294538

[26] Zhan J Wei Q Guo W Liu Z Chen S Huang Q. Evaluating the triglyceride glucose index as a predictive biomarker for osteoporosis in patients with type 2 diabetes. Front Endocrinol (Lausanne). (2025) 16:1534232. doi: 10.3389/fendo.2025.1534232, PMID: 40260282 PMC12010436

[27] Chen Y Zhang Y Qin S Yu F Ni Y Zhong J. The correlation between TyG-BMI and the risk of osteoporosis in middle-aged and elderly patients with type 2 diabetes mellitus. Front Nutr. (2025) 12:1525105. doi: 10.3389/fnut.2025.1525105, PMID: 40135223 PMC11932904

[28] Tian C Liu J Ma M Wang S Zhang Y Feng Z. Association between surrogate marker of insulin resistance and bone mineral density in US adults without diabetes. Arch Osteoporos. (2024) 19:42. doi: 10.1007/s11657-024-01395-2, PMID: 38796579

[29] Fassio A Idolazzi L Rossini M Gatti D Adami G Giollo A. The obesity paradox and osteoporosis. Eat Weight Disord. (2018) 23:293–302. doi: 10.1007/s40519-018-0505-2, PMID: 29637521

