# Supplementary material for: Association of triglyceride glucose body mass index with osteoporosis risks in a large Chinese adult cohort
Source: Front Endocrinol (Lausanne). 2025 Sep 23;16:1680775. doi: 10.3389/fendo.2025.1680775 (PMC12500458; doi:10.3389/fendo.2025.1680775)

| Missing variable names | No. of missing |
| --- | --- |
| Fasting plasma glucose | 148 |
| HbA1c | 97 |
| eGFR | 56 |
| Lipid panels | 27 |
| DXA | 287 |

Supplementary table 1 Variable‑level missingness

Supplementary table 2 Sensitivity analysis excluding participants who had been diagnosed as osteoporosis at the first six months of follow-up.

|  | No. of participants | No. of cases | Mean follow-up years | Crude model | Multiple variable adjusted model |
| --- | --- | --- | --- | --- | --- |
| TyG-BMI |  |  |  |  |  |
| Q1 | 5975 | 64 | 3.80 (1.99) | 1.00 | 1.00 |
| Q2 | 5974 | 66 | 3.92 (1.92) | 0.99 (0.70, 1.40) | 0.79 (0.56, 1.11) |
| Q3 | 5974 | 128 | 4 (1.91) | 1.86 (1.38, 2.52) | 1.20 (0.88, 1.62) |
| Q4 | 5974 | 843 | 4.11 (1.82) | 12.0 (9.30, 15.5) | 3.91 (2.95, 5.17) |

Supplementary figure 1 Restricted cubic splines for TyG‑BMI as a continuous variable


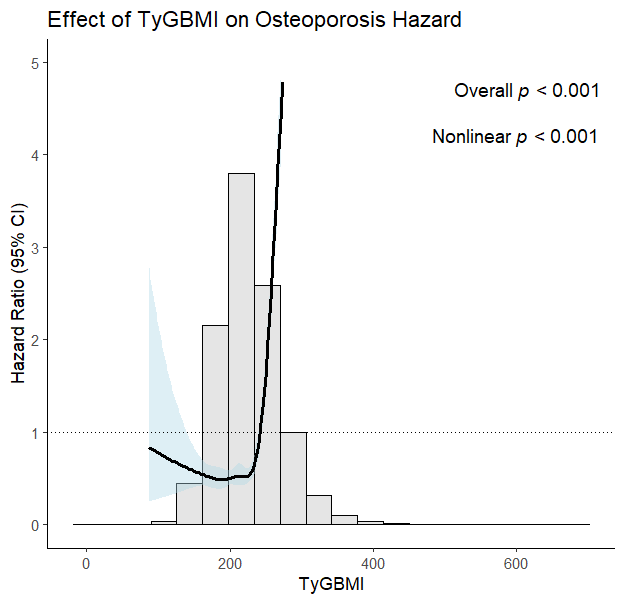

Supplement: Supplementary file 1 [file DataSheet1.docx]
